# Sleep as a moderator of adolescent brain development—evidence from a longitudinal MRI study

**DOI:** 10.1093/sleep/zsag011

**Published:** 2026-01-21

**Authors:** Salome Wild, Andrea Inderkum, Daniela Rupp, Andjela Markovic, Chiara E G Castiglione, Christoph Hamann, Kristina Adorjan, Michael Kaess, Ruth L O’Gorman Tuura, Leila Tarokh

**Affiliations:** University Hospital of Child and Adolescent Psychiatry and Psychotherapy, University of Bern, Bern, Switzerland; Graduate School for Health Sciences, University of Bern, Bern, Switzerland; Translational Research Center, University Hospital of Psychiatry and Psychotherapy, University of Bern, Bern, Switzerland; Institute of Pharmacology and Toxicology, University of Zurich, Zurich, Switzerland; University Hospital of Child and Adolescent Psychiatry and Psychotherapy, University of Bern, Bern, Switzerland; Bern University of Teacher Education, Institute of Secondary Education, Bern, Switzerland; University Hospital of Child and Adolescent Psychiatry and Psychotherapy, University of Bern, Bern, Switzerland; Translational Research Center, University Hospital of Psychiatry and Psychotherapy, University of Bern, Bern, Switzerland; Department of Psychology, University of Fribourg, Fribourg, Switzerland; University Hospital of Child and Adolescent Psychiatry and Psychotherapy, University of Bern, Bern, Switzerland; Translational Research Center, University Hospital of Psychiatry and Psychotherapy, University of Bern, Bern, Switzerland; Division of Child and Adolescent Psychiatry and Psychosomatic Medicine, Department of Pediatrics, Inselspital, Bern University Hospital, University of Bern, Bern, Switzerland; Translational Research Center, University Hospital of Psychiatry and Psychotherapy, University of Bern, Bern, Switzerland; University Hospital of Child and Adolescent Psychiatry and Psychotherapy, University of Bern, Bern, Switzerland; Department of Child and Adolescent Psychiatry, Centre for Psychosocial Medicine, University Hospital Heidelberg, Heidelberg, Germany; Center for MR Research, University Children's Hospital Zurich, University of Zurich, Zurich, Switzerland; Neuroscience Center Zurich, University of Zurich, Zurich, Switzerland; Children’s Research Centre, University Children’s Hospital Zurich, Zurich, Switzerland; University Hospital of Child and Adolescent Psychiatry and Psychotherapy, University of Bern, Bern, Switzerland; Translational Research Center, University Hospital of Psychiatry and Psychotherapy, University of Bern, Bern, Switzerland

**Keywords:** adolescence, brain development, grey matter volumes, longitudinal sleep assessment, actigraphy, MRI, moderation analysis

## Abstract

**Study objectives:**

Adolescent brain maturation is characterized by profound structural changes to grey and white matter. While cross-sectional studies in youth indicate that measures such as sleep duration and regularity are linked to regional grey matter volumes, there is less evidence on how sleep may shape developmental trajectories over time. Therefore, the goal of this study was to test whether objectively measured sleep is a moderator of grey matter volume changes in early adolescence.

**Methods:**

Structural MRI was acquired longitudinally at two time points six months apart in 39 healthy adolescents aged 10 to 14 years (mean age = 12.72 ± 1.00 years). Between the two MRI scans, we collected daily objective sleep/wake behavior using actigraphy (mean = 129.95 ± 34.01 nights). We then examined how sleep duration, efficiency, timing, and regularity moderate changes in seven regions-of-interest (ROIs) linked to social/emotional functioning using univariate simple moderation models for each ROI and sleep characteristic.

**Results:**

Sleep duration and efficiency moderated grey matter volume changes in regions including the thalamus, precuneus, orbitofrontal cortex, and amygdala (.11 ≤ Δ*R*^2^ ≥ .50; .001 < *p* <.020). Shorter and more disrupted sleep was associated with attenuated structural changes. Sleep regularity and sleep midpoint further moderated changes in grey matter volume (.05 ≤ Δ*R*^2^ ≥ .30, .001 < *p* <.043), indicating a role for these parameters in brain development.

**Conclusion:**

These findings highlight the potential role of sleep in adolescent neurodevelopment and underscore the potential for targeted interventions to support brain health during this critical window.

## Graphical Abstract

**Graphical Abstract:**
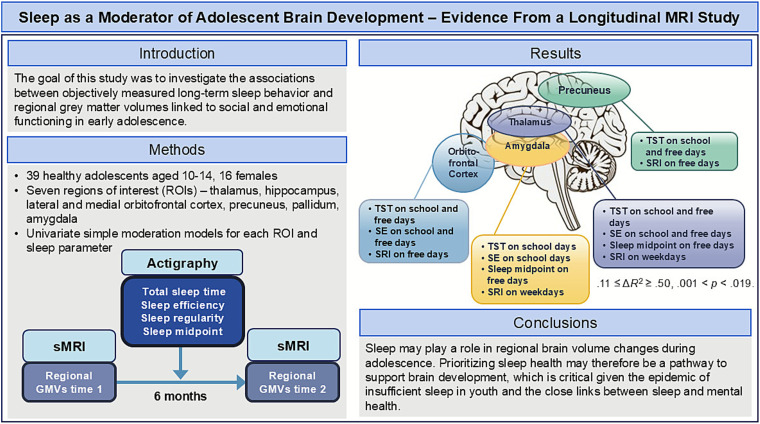

Statement of Significance Adolescence is a period of extensive brain remodeling partly influenced by modifiable behaviors; here, we identify sleep as a key moderator of brain development. Using a longitudinal design, we find that over a 6-month period, objectively measured sleep parameters including duration, timing and regularity moderate grey matter volume changes in brain regions associated with cognition and emotion regulation. Given the epidemic of insufficient sleep in youth, the close ties between sleep and mental health, and the potential of adolescence to shape long-term developmental outcomes, these findings suggest that prioritizing sleep may be a pathway to support healthy brain development. Future neuroimaging studies should include large samples and extended follow-up intervals to better capture the links between sleep and brain maturation.

## Introduction

The transition from childhood to adolescence is marked by rapid physical, social, emotional, and cognitive maturation. In parallel to these more readily observable shifts, the adolescent brain undergoes marked changes in both structure and function [1–4]. A key aspect of this restructuring is a non-linear, region-specific decline in cortical grey matter volume [5–7], concurrent with an increase in cortical white matter volume. While the decrease in grey matter volume is largely attributable to synaptic pruning [8], the increase in white matter is a consequence of myelination and an expansion of axonal diameter [9–13].

Despite identifiable general developmental pathways, individual trajectories for both cortical and subcortical structures vary considerably [14, 15]. This interindividual variability in brain maturation is in large part under genetic control. For example, a longitudinal MRI study used a twin design to disentangle genetic from environmental contributors and found that the genetic influence on white and grey matter volume in early adolescence is generally large, i.e., explains 50 to 90% of variance, with some variations across different brain regions [16]. These findings and others [17] suggest that the significant restructuring that happens during adolescence is influenced by genes in a time and region-dependent manner.

Although genetic factors play a pivotal role in adolescent brain development, environmental factors also shape these developmental trajectories. One factor that may influence the developmental trajectory of brain maturation is sleep—a building block of both physical and mental health. During adolescence, environmental factors (e.g., early school start times and nighttime electronics use) interact with biological processes (e.g., increased tolerance for sleep pressure and a shift in the circadian timing system towards later bedtimes) to compress the “sleep window,” leading to a pattern of inadequate sleep [18–22]. In fact, sleep restriction in adolescence is so common that it has been called a chronic and global issue [21, 23], with detrimental effects on health related outcomes such as cognitive functioning, emotion regulation and risk-taking behavior [22, 24, 25] to name a few.

Beyond its impact on adolescent physical and mental health, sleep restriction may also influence brain maturation. It has been argued that sleep is “by the brain and for the brain” [26]—a function that might be especially critical during adolescence. Evidence from animal studies indicates that sleep may be actively involved in shaping neuronal structure during adolescence. For example, sleep deprivation was shown to interfere with the decrease in spine density that is typically associated with healthy maturation in adolescent mice [27]. Moreover, chronic sleep deprivation was associated with cellular stress in the frontal cortex of adolescent mice [28], further highlighting the negative impact of chronic short sleep. Results from another study in which adolescent mice underwent 72-hours of sleep deprivation, indicated that sleep loss compromised maturationally appropriate synaptic pruning [29, 30], likely due to a reduction of microglial phagocytosis [31]. Moreover, in a study in which adolescent mice were sleep restricted for 14 days, neurogenesis in the hippocampus was diminished and accompanied by compromised long-term memory – an effect that persisted until early adulthood. A proposed mechanism underlying this finding may be that sleep restriction leads to alterations of astrocyte-related molecules, that is, glial cells that are implicated in the process of hippocampal neurogenesis [32]. In a similar vein, adolescent rats that underwent chronic sleep restriction for one month showed reduced hippocampal volume, however, the authors concluded that this effect likely reflects a decrease in the size of neuronal cell bodies and dendritic arborizations rather than impaired neurogenesis [33]. In sum, experimental animal studies provide evidence of the negative repercussions of short sleep for brain development, with potentially long-lasting consequences on functional outcomes such as memory or social interaction [32, 34].

While findings stemming from experimental research in rodents point to an active role of sleep in brain development, there is a growing body of evidence suggesting similar associations in humans. Several studies using structural MRI have now examined the association between grey matter volume and sleep in youth [35–37]. For example, one study [36] found that longer subjective sleep duration was positively associated with hippocampal grey matter volume in 290 children and adolescents aged 5 to 18 years. Furthermore, in a large longitudinal study of 9/10 year-olds from the Adolescent Brain Cognitive Development (ABCD) study, children with insufficient (< 9 hours per night, based on parent-report) sleep showed reduced grey matter volume in several regions as compared to those with sufficient sleep (> 9 hours) [37]. These group differences were present at both baseline and at 2-year follow-up, indicating relatively stable structural differences associated with short sleep. Similarly, in another study based on the ABCD sample with participants aged 9 to 11 years [38], short sleep (based on parent-report) was correlated with smaller volumes in cortical and subcortical structures, including but not limited to the lateral and medial orbitofrontal cortex, precuneus, posterior cingulate cortex, ventromedial prefrontal cortex, thalamus, caudate, and pallidum. In contrast to aforementioned studies [36–38], in a cross-sectional study of 9- to 25-year-olds, self-reported shorter sleep duration was linked to larger subcortical volumes in the hippocampus, pallidum and amygdala. Moreover, this study found that larger palladium and thalamus volumes were also associated with more wake time after sleep onset. These finding were interpreted by the authors as an acceleration of neurodevelopment due to stressors [35].

In addition to sleep duration and sleep continuity, sleep habits (e.g., the timing of sleep or variability in sleep duration) may also play a role in adolescent brain development. For example, in a cross-sectional study of 177 fourteen year olds, self-reported later bedtimes on weekends were associated with smaller grey matter volumes in several regions, including frontal, precuneus and anterior cingulate regions [39]. A follow-up study conducted at age 16 with 101 participants of the original sample found an association between later self-reported wake-up times on weekends and smaller grey matter volumes in the medial prefrontal cortex and the amygdala. Larger discrepancies in weekday versus weekend wake-up times were linked to reduced grey matter volumes in the right hippocampus and amygdala [40], indicating that sleep schedule regularity may influence brain structure [41]. In line with these findings, a recent longitudinal study of 963 adolescents reported that greater delays in sleep timing (measured via several days of actigraphy) from ages 10–13 to 13–16 years were associated with increased grey matter volume in the thalamus and pallidum, and decreased volume in the putamen, nucleus accumbens, and ventral diencephalon [40]. Finally, in another longitudinal study of 128 adolescents, higher self-reported eveningness at ages 14 and 19 was associated with greater grey matter volumes in the right medial prefrontal cortex [42].

Building on insights from previous human studies, this manuscript aims to advance our understanding of the role of sleep in adolescent brain development by addressing methodological limitations in earlier research. On the one hand, there is a need for more longitudinal studies that capture long-term sleep behavior in combination with repeated measures of brain structure, given the large inter-individual variability. On the other hand, findings that are based solely on self- and parent-reported sleep may have several limitations compared to those derived from objective measures; for example, they may be affected by recall bias [43, 44] as they are based on a mental average of those nights that can be remembered. Additionally, self-reports of sleep typically overestimate sleep duration [45, 46]. For instance, one study of 11- to 13-year-olds [45] found weak and non-significant correlations between objective (actigraphy) and subjective sleep duration (self-report questionnaire; r^2^ = 0.06). Moreover, prior studies often use relatively short or undefined time frames when asking about participants’ sleep, which is problematic given that sleep, as a likely long-term influence on brain development, undergoes substantial changes over time.

To bridge this gap, we examined the moderating role of objectively measured sleep on changes in grey matter volume across seven brain regions of interest (ROIs) – thalamus [38, 40], hippocampus [35, 36, 41], lateral and the medial orbitofrontal cortex [38], precuneus [38], pallidum [35, 38, 40] and amygdala [35, 41]. We selected these ROIs based on the following rationale: (1) our modest sample size prevents us from examining associations across the entire brain, (2) previous studies have found an association between these regions and sleep parameters and (3) they are part of the brain networks involved in social and emotional functioning that undergo rapid maturation during adolescence [47–49].

Alterations in the maturation of the social–emotional network contribute to mental health disorders, about 35% of which of have their onset before the age of 14 years [50, 51]. In particular, the amygdala, the lateral orbitofrontal cortex and the precuneus are part of the so called “social brain,” a network of cortex and subcortical regions that is tied to social cognition and social abilities. These skills markedly develop during the adolescent years [48, 49], and deficits in social cognition have been linked to compromised mental health [52–55]. Furthermore, the thalamus, hippocampus, amygdala, and pallidum are all integrated into the limbic system, a network that is essential for emotion regulation [56–63] – a function that is frequently impaired in psychiatric disorders such as depression [64, 65]. In parallel to impaired function, altered volumes in our ROIs have been associated with a number of psychiatric symptoms in adolescence [66–74].

Based on the evidence provided by cross-sectional studies on sleep and grey matter volumes, we hypothesized that indices of healthy sleep (i.e., longer sleep duration, higher sleep efficiency and more regular sleep–wake patterns) would facilitate normative developmental trajectories with regards to the thalamus, the lateral and medial orbitofrontal cortex, the precuneus, and the pallidum. In contrast, we hypothesized that healthier sleep would be accompanied by few volume changes in the amygdala and the hippocampus, which, unlike our other ROIs, are regions that show increases in grey matter volume during puberty [59, 75, 76]. To test these hypotheses, we measured MRI at two time points 6 months apart in a sample of healthy young adolescents between 10 and 14 years of age, while sleep in the intervening period was measured continuously using actigraphy.

## Methods

### Participants

Forty-five participants (19 female; mean age = 12.69 years, SD = 1.02 years) aged 10 to 14 years initially participated in the study. Two participants did not complete the second MRI scan and were therefore excluded from the analysis. In addition, one participant was excluded due to poor MRI image quality, two were excluded because their thalamic and intracranial volumes were identified as outliers upon visual inspection, and one participant was excluded due to artifacts in the MRI caused by dental braces. Therefore, MRI and actigraphy data of 39 healthy subjects (16 females; mean age = 12.72; SD ±1.00) were analyzed. Participants were recruited for a twin study focusing on the heritability of sleep and its impact on brain development [77–81]. The sample consisted of twelve monozygotic, seven dizygotic twin pairs and one additional “single” participant whose monozygotic twin was excluded because no follow-up MRI scan was available. As indicated by self-reported pubertal development ratings reflecting Tanner stages [82] at baseline, the sample consisted of 20 pre/early pubertal (Tanner 1: 6, Tanner 2: 14), 7 mid pubertal (Tanner 3), and 12 late/post pubertal (Tanner 4: 11, Tanner 5: 1) participants. When comparing self-reported pubertal stage at baseline to the stage reported at follow-up, the majority (26 of 39) of participants did not change pubertal status. Of those who did (10 males, three females), 10 participants advanced one stage and three participants two stages.

Exclusion criteria included the presence of any chronic or acute physical illness, a diagnosis of any neurodevelopmental or neuropsychiatric disorder, the use of medications affecting sleep or brain function, and premature birth (i.e., birth before 30 weeks of gestation). Furthermore, young adolescents with sleep disorders, excessive daytime sleepiness, or frequent napping were not eligible to participate in the study. Exclusion criteria were evaluated using information from both self- and parent-reports. Written informed consent was obtained from all participants and their parents, and the study was approved by the local ethics committee in accordance with the Declaration of Helsinki.

### Procedure

Daily sleep behavior was captured by watch-like, wrist worn micro-mechanical triaxial accelerometers (Jawbone UP move) for six months. These accelerometers, called actigraphs, allow for the delineation of sleep–wake patterns based on motion. MRI scans were performed at the start of the study and at completion of the six-month period to evaluate brain maturation. The actigraphy devices were designed to be worn 24 hours a day and 7 days a week on the non-dominant hand. No instructions on sleep behavior were given for the six months of study participation, meaning that bed and rise times were freely determined by the participants and their parents. Sleep parameters were derived using Jawbone’s proprietary algorithm and all data were carefully inspected. Each night was visually reviewed individually, and nights were flagged for potential exclusion when rest–activity patterns suggested device error (e.g., accidental switching between sleep and active mode), when bed or rise times were unusually early or late, or when total sleep time was implausibly short or long. Nights were excluded if the activity pattern confirmed an artefact, such as no activity following the recorded rise time. To guarantee continuous recording of sleep–wake behavior, the batteries of the participants’ actigraphs were replaced as soon as they ran low. To support the correct identification of sleep and wake periods, participants were instructed to press a button on the device at lights-out in the evening and upon walking in the morning, which caused the device to switch from the” active mode” to the “sleep mode.” If the button was not pressed and hence, the event markers were missing, the data was not included in the analysis.

### Sleep assessment

The following parameters were derived from the actigraphs with minute precision: sleep duration (total sleep time [TST]), sleep start time, sleep end time, time in bed (TIB), wake after sleep onset (WASO) and sleep onset latency (SOL). A prior study in a clinical cohort of children and adolescents [83] indicated good agreement between the actigraphs used in this study and polysomnography-derived variables [84]. In order to reduce multiple testing, we used sleep efficiency (SE) defined as the ratio of total sleep time to TIB [85] since this measure integrates SOL and WASO. Furthermore, sleep midpoint, a marker of the circadian system, was calculated as the midpoint (in clock time) between sleep onset and offset [86, 87]. Lastly, to assess the regularity of sleep schedules, the Sleep Regularity Index (SRI) was calculated [88]. The SRI quantifies the likelihood of an individual being in a congruent state, that is, either awake or asleep at any given time-point (minute-by-minute) and 24 hours apart. Regularity values derived from the SRI can theoretically vary between 0 and 100, with a score of 100 indicating perfect regularity, while a score of 0 would reflect arbitrary sleep–wake-patterns [88]. Therefore, we examined the following sleep variables for the current analysis: TST, Sleep Efficiency (SE), Sleep Midpoint and SRI.

The mean and standard deviation of all sleep parameters were calculated separately for school and free days for each participant. Free days included weekends, public holidays and school holidays (for sleep regularity, free days corresponded to weekends and school holidays). Sleep on school days included nights for which the participants had to go to school the following morning, while sleep on free days corresponded to those nights on weekends and during holidays that were not followed by school the next morning. The distinction between free days and school days for TST, SE, and SRI is crucial because sleep duration on free days is longer, while bed and risetimes are later [89–92]. Of note, for Sleep Midpoint we further analyzed data from free days (corresponding to weekends and holidays) only, because we were interested in the circadian timing system which is most reliably captured in the absence of environmental factors such as school schedules. Rest and activity patterns of each night were visually inspected to ensure data quality under consideration of criteria applied in prior studies [77, 93]. After visual inspection, 3.58% (278 nights) were excluded from the analysis. In total, 4938 nights were analyzed, from which 2’272 (46% of all nights) were free nights and 2666 (54% of all nights) were labeled as school nights. Of all free nights, 1049 (46%) were weekend nights and 1223 (54%) were nights during holidays. On average, 130 nights (± 34 nights; about four months of data) per participant were available. Due to variability in compliance, the number of measured nights per subject ranged from 50 to 192 nights. Across all subjects, data from 2560 nights were excluded because the actigraph was not worn (range: 15 – 149 nights). Data for this study were collected across the entire year and were evenly distributed across different seasons.

### Structural MRI data

Participants underwent scanning on a 3.0 T MRI scanner (GE Medical Systems MR750) located at the University Children’s Hospital Zurich, Switzerland, using an eight-channel head coil to acquire T1-weighted three-dimensional fast inversion-recovery prepared spoiled gradient recalled (IR-SPGR) images (Inversion time (TI) = 600 ms, echo time (TE) = 4.25 ms, repetition time (TR) = 11.4 ms, flip angle = 8°, and isotropic voxel resolution (1 mm)). Subjects were familiarized with the scanning environment prior to image acquisition and instructed to stay awake during the entire scanning procedure, which was ensured by talking to the subjects between each scan. Structural images were assessed for quality and if evaluated as poor, the scan was repeated.

Volumetric segmentation and cortical reconstruction were performed using FreeSurfer (FS; Version 5.3 [94]), an open-source, fully automated brain image morphometry software package. MRI scan quality was assessed visually prior to processing of the data through the automated FS pipeline. All images were rated as poor, good, or very good, and the T1 images rated as poor quality (e.g., due to excessive movement or other artifacts) were not used for analysis. The brain MRI images underwent several standard, automated image processing steps to achieve brain parcellation. The processing procedure (implemented by means of the “recon-all” script using the default analysis settings) included skull-stripping [95], intensity normalization [96], subcortical segmentation [97, 98], surface generation [99], topology correction [100, 101], surface inflation [102], registration to a spherical atlas [103], and thickness calculation [104].

Following automated processing, appropriate segmentation was ensured by visual inspection of all processed images. No manual corrections were deemed necessary for the automated segmentation results. Intracranial volume (ICV) was estimated using an automated procedure in which the volume-scaling factor was derived by registration of each individual to an atlas template, representing the image atlas of Talairach and Tournoux (1988) [105].

The intracranial volume estimated for each participant was then used to correct for inter-individual variability in head size using the proportion approach [106–109]. This method is commonly used to normalize regional brain volumes [110, 111]. In line with this approach, all regional grey matter volumes which were extracted from FS statistics (“aseg.stats” files for subcortical structures and “aparc.stats” files for cortical structures) were divided by ICV [106, 112], which resulted in a ratio between 0 and 1 for each region of interest. All analyses presented herein were conducted using these adjusted values The total volumes of our ROIs (thalamus, hippocampus, lateral and medial orbitofrontal cortex, precuneus, pallidum, and amygdala) were calculated based on the total lateral volumes provided for each of the brain regions studied. For each of these structures, we tested whether sleep may have a moderating influence on volume change.

### Statistical analyses

All statistical analyses were performed using the IBM SPSS statistical software package (version 28) and the PROCESS macro for SPSS^113^.

### Descriptive analyses

To describe our sample, the mean and standard deviation from the actigraphy-derived sleep variables were calculated for both school- and free days for each participant. The impact of day type (i.e., school vs. free day), sex, and baseline pubertal stages on the four sleep parameters of interest (TST, SE, sleep midpoint and SRI) was examined using mixed model ANOVA. Also, a mixed model ANOVA with between-subjects factor sex (female versus male) and within-subjects factor time (baseline versus follow-up) was used to assess sex and maturational effects on the MRI measured ROIs. For this first set of analyses aimed at characterizing our sample, we applied the Holm–Bonferroni method to correct for multiple comparisons, an approach that provides strong family-wise error rate control [113, 114].

### Primary hypothesis tests

Reflecting our main research question, we aimed to determine whether sleep within the 6-month period between the baseline and follow up structural brain imaging sessions moderated the change in grey matter volumes. To test this hypothesis, simple moderation models in PROCESS [115] were used. These models consist of one predictor and one outcome variable, with a third variable specified as the moderator. To test for a moderation effect, an interaction term (arising from the predictor and the moderator variable) is added to the model. If an interaction is significant, conditional effects at specific values of the moderator can be examined to assess whether the relationship between the predictor and outcome varies by level of the moderator [115]. In line with this model, a moderating influence of a single sleep variable (e.g., Total Sleep Time) on the selected brain volumes of interest (e.g., Thalamus) over time was specified, with baseline and follow-up volumes serving as predictor and outcome, respectively. In other words, for each brain region, a separate moderation model was tested and each sleep variable measured on both free and school days (except for sleep midpoint, which was only measured on free days) was tested for a moderating effect. Sex and self-reported pubertal status (Tanner stage) [82] at baseline were included as covariates to control for potential confounds in each analysis. Age was not introduced as a separate covariate since all volumes were adjusted for ICV, which serves as a proxy for biological age [116]. Furthermore, pubertal status was included in all models, providing another metric of maturation. Since we chose to investigate seven ROIs and four sleep variables (on free days and on school days for TST, SE, and SRI, and on free days for sleep midpoint), a total of 49 analyses were run.

In view of the modest sample size (n = 39), multivariate approaches that would have considered all our ROIs and sleep variables simultaneously or include subsets of conceptually related variables (e.g., functionally or anatomically related structures) were not deemed appropriate due to the large number of parameters relative to observations. Therefore, we tested moderation effects in a series of separate univariate models for each regional volume and sleep variable separately for free days and weekdays. This approach provides more stable parameter estimates as compared to multivariate models in a small sample while allowing for region-specific interpretation of effects. The Benjamini-Hochberg False Discovery Rate (FDR)–procedure [117] was used across all models to correct for multiple comparisons and thereby control for the proportion of false positives. We chose to use the FDR-approach for examining our primary hypothesis because we ran a relatively large number of tests (49) for which procedures such as the Bonferroni-Holm correction may have been too conservative [114]. Moreover, the FDR-approach has been used in several studies of sleep and brain development [35, 38, 118].

### Post hoc evaluation of conditional effects

Lastly, to further assess the moderating effects of our a-priori defined sleep variables (TST, SE, sleep regularity and sleep midpoint) on changes in brain volume within our ROIs, we analyzed the conditional effects for those models for which a significant interaction had emerged. Specifically, the conditional effects at the mean, one standard deviation below, and one standard deviation above the mean for each sleep variable were examined. Given the specification of predictor and outcome variables as grey matter volumes at baseline and follow-up (six months apart) respectively, the analysis of simple slopes of the moderator (i.e., sleep variable) was used to assess the relationship of grey matter volumes over time. Larger associations between the two time points (e.g., larger b-values) were interpreted as indicative of smaller changes (i.e., more stability) between baseline and follow-up volumes. In turn, a weaker relationship between baseline and follow-up volumes (i.e., smaller b-values) were interpreted as indicators of more volume change over time. Additionally, differences between the simple slopes were assessed using direct contrast analysis [119], that is, a comparison between the confidence intervals of the simple slopes to determine whether conditional effects were significantly different from each other. Overlapping confidence intervals were interpreted as indicating non-significant differences between simple slopes.

## Results

### Descriptive analyses

Table 1 shows mean and standard deviations for the four actigraphy-derived sleep parameters of interest—TST, SE, SRI, and sleep midpoint. Type of day (free or school day) and sex are shown separately for each parameter. For the most part, the participants in our sample were good sleepers, with sleep efficiency above 85%. As expected, total sleep time was longer (*p* **=** .012) and timing was later (*p* **=** .012) on free as compared to school days (see Table 1). Sleep was more regular on school days than on weekends and school holidays (*p* **=** .012). No sex differences for any of the sleep variables or interaction effects between day of measurement (school vs. free) and sex were found. Similarly, there was no main effect of pubertal stage on any of our four sleep parameters (i.e., TST, SE, sleep midpoint, SRI), meaning that we could not find systematic variation in sleep behavior between different stages of puberty. Moreover, we found no significant interaction effects between pubertal stage and type of day (that is, sleep on school vs. sleep on free days) for any of our sleep variables, indicating that differences between school and free days did not vary as a function of pubertal stage.

**Table 1:** Overview of actigraphy derived sleep parameters

	**School day**			**Free day**			**School vs Free day (day type)**	**Sex**	Interaction sex^*^day type	**Pubertal status (t1)**	Interaction pubertal status^*^day type
**Phenotype**	**Male**	**Female**	**Total**	**Male**	**Female**	**Total**					
TST (minutes)	492 ± 26	489 ± 37	491 ± 30	507 ± 25	523 ± 31	514 ± 29	*F*(32.79), *p* = .012	*F*(0.566), *p* > .999	*F*(5.3), *p* = .243	*F*(0.794), *p* > .999	*F*(1.82), *p* = .917
SE (%)	90.41 ± 2.75	91.53 ± 3.23	90.87 ±2.97	90.51 ± 2.96	92.34 ± 2.59	91.26 ± 2.93	*F*(2.166), *p* > .999	*F*(2.767), *p* = .84	*F*(1.365), *p* > .999	*F*(2.346), *p* = .592	*F*(0.278), *p* > .999
Sleep mid-point (h)	2:29 a.m. ± 0.35	2:22 AM ± 0.51	2:26 AM ±0.42	3:35 AM ± 0.68	3:45 AM ± 0.68	3:48 AM ± 0.67	*F*(316.297), *p* = .012	*F*(0.397), *p* > .999	*F*(0.021), *p* > .999	*F*(1.911), *p* = .917	*F*(0.924), *p* > .999
SRI	91.16 ± 2.08	91.21 ± 3.45	91.18 ± 2.70	84.05 ± 2.94	84.52 ± 4.88	84.25 ± 3.83	*F*(133.067)*,* *p* = .012	*F*(0.079), *p* > .999	*F*(0.128), *p* > .999	*F*(1.486), *p* > .999	*F*(0.447), *p* > .999

Mean and standard deviations of sleep parameters used in the study are shown as rows. Values are shown separately for school days, defined as nights where the participants had to go to school the following morning, and free days, defined as weekends and holidays. The table shows F- and *p*-values from the mixed model ANOVA with within-factor day (school versus free) and between-factors sex (male versus female) or pubertal stage as well as the interaction terms. Significant values (*p* < .05) are highlighted in bold (n = 39). Correction for multiple comparisons was applied using the Holm-Bonferroni method. TST (total sleep time), SE (sleep efficiency) and SRI (Sleep Regularity Index).

Results of mixed model ANOVA comparing time 1 and time 2 grey matter volumes are shown in Table 2. No significant differences in ICV corrected volume between the time 1 and time 2 MRI sessions were found. There were no significant sex differences with regards to grey matter volumes, nor significant interactions between time and sex.

**Table 2:** Statistical analysis of brain volume changes as a function of time and sex and their interaction

	**T1 vs T2**	**Male vs Female**	Interaction
Phenotype			
Thalamus	F(1.563), *p* > .999	F (2.064), *p* > .999	F(1.058), *p* = > .999
Hippocampus	F(7.273), *p* = .21	F (0.131), *p* > .999	F(0.280), *p* > .999
Lateral Orbitofrontal Cortex	F(0.132), *p* > .999	F (3.271), *p* > .999	F (0.667), *p* > .999
Medial Orbitofrontal Cortex	F(0.050), *p* > .999	F(0.849), *p* > .999	F(0.032), *p* > .999
Precuneus	F(1.141), *p* > .999	F(5.989), *p* = .38	F(0.515), *p* > .999
Pallidum	F(0.045), *p* > .999	F(0.804), *p* > .999	F(0.052), *p* > .999
Amygdala	F(1.226), *p* > .999	F(0.873), *p* > .999	F(0.043), *p* > .999

The comparison of brain volumes measured at baseline (T1) and six months later at follow-up (T2) is shown in the first column, while the comparison between sex, that is, male and female, is shown in the second column. The table further depicts the interaction between time and sex, that is, whether differences in changes in brain volumes over time are predicted by sex. F- and *p*-values shown here stem from a mixed model ANOVA with time of measurement (time 1 versus time 2) as a within-subjects and sex (male versus female) as a between-subjects factor. Correction for multiple comparisons was applied using the Holm-Bonferroni method, n = 39.

### Primary hypothesis tests and post hoc evaluation of conditional effects

**Figure 1:**
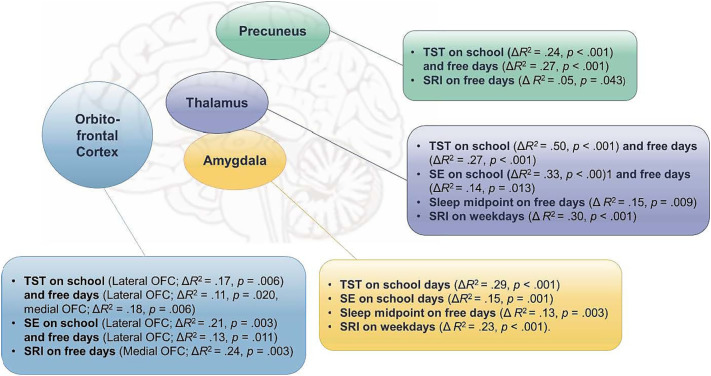
Sleep variables moderating changes in brain volumes. Schematic depiction of the regions of interest for which sleep moderated volume changes over time. Regions of interest were the thalamus, precuneus, the lateral and medial orbitofrontal cortex, the hippocampus, pallidum and the amygdala. Sleep variables were measured on school days and on free days. School days included nights for which the participants had to go to school the following morning, while free days corresponded to nights on weekends and during holidays. TST: Total sleep time, SE: Sleep efficiency, SRI: Sleep regularity index, *ΔR*^2^: Effect size of the moderation. Image (schematic depiction of the brain) used under license from Adobe Stock/Oleksandr Pokusai.

In our moderation analyses, we found that all the sleep variables studied had significant moderating effects on changes in grey matter volume. Figure 1 illustrates the brain regions of interest and the sleep variables that emerged as significant moderators of volume change, while more detailed statistics of the significant moderation models can be found in Table 3, including conditional effects at different values of the moderator variable (e.g., effects at mean TST and at TST ± 1 SD from the mean) and an interpretation of the findings. Larger conditional effects were interpreted as indicative of a stronger association, i.e., *smaller* volume change between time 1 and time 2 grey matter volumes. Furthermore, Figures S1–S7 in the supplementary material depict the conditional effects (simple slopes) for each model that showed significant moderation effects, reflecting the relationship between baseline and follow-up volumes at different levels of the moderator (sleep) variables.

**Table 3:** Overview of significant interaction effects and conditional effects (CE) in the moderation models tested

Moderator variable	ROI	Value of moderator for CE	*b*	*SE b*	*t*	*p*	Δ*R*^*2*^	95% CI for CE	Interpretation of moderating effect based on simple slopes
TST school days									
Interaction effect	Thalamus		−1.18	.14	−8.55	< .001	.50		Sleep duration below the mean was associated with smaller volume change as compared to mean sleep duration.
Conditional effects at values of the moderator		Below mean (7.67 h) Mean (8.18 h) Above mean (8.69 h)	1.28 0.68 0.07	.15 .10 .10	8.55 6.45 0.76	< .001 < .001 .450		[0.97, 1.58] [0.46, 0.89] [−0.12, 0.27]	
Interaction effect	Precuneus		−.61	.09	−6.49	< .001	.24		Sleep duration below the mean was associated with smaller volume change as compared to above mean sleep duration.
Conditional effects at values of the moderator		Below mean (7.67 h) Mean (8.18 h) Above mean (8.69 h)	0.96 0.64 0.33	.11 .07 .06	8.89 8.80 5.41	< .001 < .001 < .001		[0.74, 1.18] [0.50, 0.79] [0.21, 0.46]	
Interaction effect	lOFC		−.63	.29	−3.02	.006	.17		No significant differences in volume change between the three different levels of sleep duration (above, below and at the mean) were found.
Conditional effects at values of the moderator		Below mean (7.67 h) Mean (8.18 h) Above mean (8.69 h)	0.81 0.48 0.16	.26 .17 .11	3.05 2.83 1.53	.005 .008 .134		[0.27, 1.34] [0.14, 0.83] [−0.05, 0.38]	
Interaction effect	Amygdala		1.44	.21	6.84	< .001	.29		Sleep duration above the mean was associated with smaller volume change as compared to mean sleep duration.
Conditional effects at values of the moderator		Below mean (7.67 h) Mean (8.18 h) Above mean (8.69 h)	−0.04 0.70 1.43	.21 .15 .14	−0.17 4.72 9.98	.865 < .001 < .001		[−0.47, 0.40] [0.40, 1.00] [1.14, 1.72]	
TST free									
Interaction effect	Thalamus		−1.35	.32	−4.18	< .001	.27		Sleep duration below the mean was associated with smaller volume change as compared to sleep duration above the mean.
Conditional effects at values of the moderator		Below mean (8.09 h) Mean (8.56 h) Above mean (9.05 h)	0.92 0.28 −0.37	.22 .14 .19	4.26 2.03 −1.89	< .001 .050 .067		[0.48, 1.36] [0.00, 0.55] [−0.76, 0.03]	
Interaction effect	lOFC		−.055	.22	−2.46	.020	.11		No significant differences in volume change between the three different levels of sleep duration (above, below and at the mean) were found.
Conditional effects at values of the moderator		Below mean (8.09 h) Mean (8.56 h) Above mean (9.05 h)	0.47 0.23 −0.52	.22 .14 .10	2.10 1.52 −0.52	.043 .138 .609		[0.02, 0.93] [−0.07, 0.49] [−0.26, 0.15]	
Interaction effect	mOFC		−0.67	.22	−3.04	.006	.18		Sleep duration above the mean was associated with greater volume change as compared to sleep duration below the mean.
Conditional effects at values of the moderator		Below mean (8.09 h) Mean (8.56 h) Above mean (9.05 h)	0.77 0.45 0.13	.20 .12 .10	3.86 3.83 1.32	.001 .001 .195		[0.36, 1.18] [0.21, 0.69] [−0.07, 0.34]	
Interaction effect	Precuneus		−0.73	.10	−7.44	< .001	.27		Sleep duration below the mean was associated with smaller volume change as compared to mean and above mean sleep duration.
Conditional effects at values of the moderator		Below mean (8.09 h) Mean (8.56 h) Above mean (9.05 h)	0.92 0.58 0.23	.09 .06 .06	10.01 9.27 3.87	< .001 < .001 .001		[0.74, 1.11] [0.45, 0.71] [0.12, 0.35]	
SE school days									
Interaction effect	Thalamus		−0.21	.04	−5.27	< .001	.33		For SE below the mean, baseline and follow up volumes showed a positive association, which differed from SE above the mean, where the association between baseline and follow-up thalamic volumes was negative.
Conditional effects at values of the moderator		Below mean (87.90%) Mean (90.87%) Above mean (93.83%)	1.08 0.44 −0.19	.20 .13 .14	5.38 3.55 −1.36	< .001 .001 .183		[0.67, 1.49] [0.19, 0.70] [−0.48, 0.10]	
Interaction effect	lOFC		−0.13	.04	−3.55	.003	.21		For SE below the mean, baseline and follow up volumes showed a positive association, which differed from SE above the mean, where the association between baseline and follow-up volumes was negative.
Conditional effects at values of the moderator		Below mean (87.90%) Mean (90.87%) Above mean (93.83%)	0.67 0.29 −0.10	.21 .12 .37	3.24 2.33 −0.91	.003 .026 .368		[0.25, 1.09] [0.04, 0.54] [−0.31, 0.12]	
Interaction effect	Amygdala		0.19	.05	3.87	< .001	.15		Sleep efficiency below the mean was associated with greater volume change as compared to above-mean sleep efficiency.
Conditional effects at values of the moderator		Below mean (87.90%) Mean (90.87%) Above mean (93.83%)	0.54 1.10 1.65	.24 .18 .22	2.25 6.14 7.55	.03 < .001 < .001		[0.05, 1.03] [0.73, 1.46] [1.20, 2.10]	
SE free days									
Interaction effect	Thalamus		−0.19	.07	−2.68	.013	.14		For SE below the mean, baseline and follow up volumes showed a positive association, which differed significantly from SE above the mean, where the association between baseline and follow-up thalamic volumes was negative.
Conditional effects at values of the moderator		Below mean (88.33%) Mean (91.26%) Above mean (94.19%)	0.85 0.30 −0.24	.28 .15 .23	3.04 1.98 −1.05	.005 .057 .30		[0.28, 1.41] [−0.01, 0.62] [−0.70, 0.22]	
Interaction effect	lOFC		−0.15	.05	−2.77	.011	.13		For SE below the mean, baseline and follow up volumes showed a positive association, which differed significantly from SE above the mean, for which the association between baseline and follow-up volumes was negative.
Conditional effects at values of the moderator		Below mean (88.33%) Mean (91.26%) Above mean (94.19%)	0.51 0.08 −0.36	.21 .10 .16	2.43 0.78 −2.24	.021 .439 .032		[0.08, 0.94] [−0.13, 0.28] [−0.68, -0.03]	
Sleep midpoint free days									
Interaction effect	Thalamus		0.73	.26	2.85	.009	.15		For sleep midpoint below the mean, that is, an earlier than average sleep midpoint, baseline and follow up volumes showed a negative association, which differed significantly from sleep midpoint above the mean (i.e., later than average sleep midpoint), where the association between baseline and follow-up volumes was positive.
Conditional effects at values of the moderator		Below mean (03:08 a.m.) Mean (03:48 a.m.) Above mean (04:29 a.m.)	−0.17 0.32 0.81	.20 .17 .27	−0.85 1.96 2.97	.400 .060 .006		[−0.57, 0.23] [−0.01, 0.66] [0.26, 1.37]	
Interaction effect	Amygdala		−0.97	.29	−3.40	.003	.13		Sleep midpoint below the mean, (i.e., earlier than average sleep midpoint) was associated with smaller volume change as compared to above mean, that is, later than average, sleep midpoints.
Conditional effects at values of the moderator		Below mean (03:08 a.m.) Mean (03:48 a.m.) Above mean (04:29 a.m.)	1.64 0.99 0.34	.23 .18 .29	7.03 5.48 1.19	< .001 < .001 .240		[1.17, 2.12] [0.62, 1.36] [−0.25, 0.93]	
SRI weekdays									
Interaction effect	Thalamus		−0.26	.06	−4.55	< .001	.30		For sleep regularity below the mean, baseline and follow up volumes showed a positive association, which differed significantly from sleep regularity above the mean, for which the association between baseline and follow-up volumes was negative.
Conditional effects at values of the moderator		Below mean (88.48) Mean (91.18) Above mean (93.88)	1.19 0.47 −0.24	.27 .16 .17	4.43 2.93 −1.40	< .001 .006 .171		[0.64, 1.74] [0.15, 0.80] [−0.59, 0.12]	
Interaction effect	Amygdala		0.32	.06	5.30	< .001	.23		Sleep regularity at the mean was associated with more volume change as compared to SRI above the mean.
Conditional effects at values of the moderator		Below mean (88.48) Mean (91.18) Above mean (93.88)	0.12 0.98 1.83	.25 .16 .20	0.49 6.21 9.12	.629 < .001 < .001		[− 0.38, 0.62] [0.66, 1.30] [1.42, 2.24]	
SRI free days									
Interaction effect	mOFC		0.10	.03	3.43	.003	.24		Sleep regularity below the mean was associated with more volume change as compared to sleep regularity above the mean.
Conditional effects at values of the moderator		Below mean (80.42) Mean (84.25) Above mean (88.07)	0.04 0.42 0.80	.12 .11 .20	0.40 3.74 4.06	.693 .001 < .001		[− 0.17, 0.26] [0.19, 0.65] [0.40, 1.20]	
Interaction effect	Precuneus		0.04	.02	2.11	.043	.04		No significant differences were found between the simple slopes at below mean, above mean and mean values of sleep regularity. Descriptively, higher sleep regularity seemed to be associated with more stability of precuneus volumes over time.
Conditional effects at values of the moderator		Below mean (80.42) Mean (84.25) Above mean (88.07)	0.28 0.45 0.62	.10 .10 .14	2.77 4.70 4.32	.009 < .001 < .001		[0.08, 0.49] [0.26, 0.65] [0.33, 0.91]	

Sleep variables were conceptualized as moderators influencing the relationship between various brain regions, measured six months apart. The table depicts all the models in which the overall model was significant (i.e., *p* < .05) and one of the sleep variables was shown to have a moderating effect on the associations of brain volumes over time, meaning that a significant interaction effect emerged. Interaction effects presented here correspond to the interactions arising from the time 1 brain volume used as predictor and the sleep variable specified as moderator in the model (e.g., Thalamus volume t1^*^TST school days). Interaction effects were adjusted for multiple comparisons using FDR-correction (Benjamini-Hochberg-approach). Sex and stages of puberty were included as covariates in each model. The examination of conditional effects (simple slopes) was done to study the relationship of brain volumes over time at different levels of the moderator, that is, at mean values and at values one SD below and above the mean for each sleep variable. TST: Total Sleep Time, SE: Sleep Efficiency, SRI: Sleep Regularity Index; ROI: Region of interest; mOFC: Medial Orbitofrontal Cortex, lOFC: Lateral Orbitofrontal Cortex; *b*: unstandardized regression weight, *SE b:* standard error for the unstandardized regression weight, *ΔR*^2^: Effect size of the moderation (i.e., the amount of additional variance explained due to the inclusion of the interaction term into the model); CI: Confidence Interval.

TST on school days moderated changes in brain volume in the thalamus (Δ*R*^2^ = .50, *p* < .001), precuneus (Δ*R*^2^ = .24, *p* < .001), lateral orbitofrontal cortex (Δ*R*^2^ = .17, *p* = .006) and amygdala (Δ*R*^2^ = .29, *p* < .001) as shown in Figures 1 and S1 and described in more detail in Table 3. This means that the addition of the interaction terms, arising from the volumes of the thalamus, precuneus, lateral OFC and the amygdala measured at time 1 (each of which served as an independent variable) and total sleep time on school days (used as moderator variable), significantly improved the model. For the thalamus and precuneus, longer sleep durations were associated with larger changes in brain volume. For the lateral orbitofrontal cortex, there were no significant differences in structural changes at the different levels of sleep duration investigated. In contrast to the thalamus and the precuneus, amygdala volumes showed larger changes at below mean sleep duration as compared to above mean sleep duration. Thus, we found relative stability of amygdala volumes over time for sleep duration above the mean.

TST on free days moderated changes in brain volume in the thalamus (Δ*R*^2^ = .27, *p* < .001), lateral orbitofrontal cortex (Δ*R*^2^ = .11, *p* = .020), medial orbitofrontal cortex (Δ*R*^2^ = .18, *p* = .006), and precuneus (Δ*R*^2^ = .27, *p* < .001) as shown in Figures 1 and S2 and summarized in Table 3. For these regions, smallest changes between time 1 and time 2 volumes were associated with free day sleep durations below the mean. Like the findings for TST on school days for thalamic and precuneus volumes, below-mean sleep duration on free days was linked to smaller volume changes in these structures. The same pattern was found for the medial orbitofrontal cortex, for which smaller volume changes were associated with shorter sleep duration (i.e., TST below the mean).

**Sleep efficiency (SE) on school days** moderated changes in brain volume in the thalamus (Δ*R*^2^ = .33, *p* < .001), lateral orbitofrontal cortex (Δ*R*^2^ = .21, *p* = .003), and amygdala (Δ *R*^2^ = .15, *p* = .001) as illustrated in Figures 1 and S3 and presented in summary in Table 3. For the thalamus and the lateral orbitofrontal cortex, the smallest changes in volume between the two assessments were found at below-mean sleep efficiency. This may point to an association of low sleep efficiency with smaller volume changes in these two structures. In comparison to thalamic and lateral orbitofrontal cortex volumes, a reverse pattern with regards to size of effects emerged for the amygdala. Specifically, higher SE was associated with greater stability of amygdala volumes over six months as compared to below-mean sleep efficiency, thus suggesting smaller volume changes for higher sleep efficiency.

**Sleep efficiency (SE) on free days** moderated changes in the thalamus (Δ*R*^2^ = .14, *p* = .013) and lateral orbitofrontal cortex (Δ*R*^2^ = .13, *p* = .011, see Figures 1 and S4 for illustration and Table 3 for a summary of statistics). As with SE on school days, the smallest change in thalamic and lateral orbitofrontal cortex volumes over time was associated with comparatively low levels of sleep efficiency.

**Sleep midpoint on free days** moderated changes in the thalamus (Δ *R*^2^ = .15, *p* = .009) and in the amygdala (Δ *R*^2^ = .13, *p* = .003; Figures 1, S5, and Table 3 for supporting statistics). With regards to thalamic volume, later circadian timing was associated with less change in volume over time. In contrast to the pattern found for the thalamus, for the amygdala, results pointed towards an association between later sleep timing and more changes in volume, however, the conditional effect for later sleep timing (i.e., for above average midpoints) did not reach statistical significance.

**Sleep regularity (SRI) on weekdays** moderated changes in brain volume in the thalamus (Δ *R*^2^ = .30, *p* < .001) and in the amygdala (Δ *R*^2^ = .23, *p* < .001) as presented in Figures 1 and S6 and further outlined in Table 3. With regards to the thalamus, sleep regularity below the mean was associated with less volume change as compared to sleep regularity above the mean*.* For the amygdala, sleep regularity above the mean was associated with smaller volume change over time as compared to mean SRI values. This may suggest higher stability of amygdala volumes with more regular sleep schedules on weekdays, contrasting the finding for the thalamus, where irregularity seemed to be linked to stability of volumes over time.

**Sleep regularity (SRI) on free days** moderated changes in brain volume in the medial orbitofrontal cortex (Δ *R*^2^ = .24, *p* = .003) and in the precuneus (Δ *R*^2^ = .05, *p* = .043; Figures 1 and S7; Table 3). For the medial orbitofrontal cortex, baseline volumes predicted follow-up volumes at both mean and above-mean SRI, with the effect strengthening as sleep regularity increased. This suggests that more regular sleep on free days may be linked to greater stability in volume over time, though the difference between mean and above-mean SRI was not statistically significant. A similar trend was observed for the precuneus, where follow-up volumes were significantly predicted by baseline volumes at all three levels of SRI (mean, one standard deviation below mean, and one standard deviation above mean). There was a gradual increase in predictive strength with higher sleep regularity, which may suggest that more consistent sleep timing on free days is associated with more stable precuneus volumes.

## Discussion

Our unique longitudinal design, integrating neuroimaging with objective long-term monitoring of sleep behavior, provides new insights into the relationships between sleep behavior and adolescent brain development. By bridging the gap between animal and cross-sectional studies reliant on subjective sleep measures in humans, we extend the understanding of sleep’s role in neurodevelopment [35–37, 39, 41]. Our findings indicate that sleep duration, quality, and timing in early adolescence may contribute to structural changes in key brain regions involved in social cognition and emotion regulation. Given the sensitivity of this developmental period to lifelong brain architecture [120], these results suggest that sleep is of critical importance for adolescent brain health. Below, we discuss the findings for each sleep variable investigated in our study.

### Sleep duration

Sleep duration moderated grey matter volume changes in all ROIs studied, except for the hippocampus and pallidum. This was true for both sleep duration on school and free days (apart from the amygdala where the effect was limited to school days). The analysis of conditional effects at different levels of total sleep time indicated that lower total sleep time was associated with smaller changes in volume in the precuneus, thalamus and the orbitofrontal cortex. These findings based on objective measures of sleep are in line with evidence from prior cross-sectional and self-reported studies linking sleep duration to grey matter volumes [35, 36, 38]. For example, a large study taking a whole brain approach in participants aged 9 to 11 years [38] also found that short sleep duration (based on parent-report) was linked to smaller precuneus, thalamic and orbitofrontal cortex volumes. Their whole brain approach also identified associations between sleep duration and volumes of the prefrontal and temporal cortex and the supramarginal gyrus, which we may have missed due to our ROI approach.

In view of the potential influence of sleep duration on brain maturation, it seems crucial to consider that there is a strong environmental influence on sleep duration measured on school days, which accounts for approximately 80% of the variance, whereas on free days, genetic factors play a more substantial role [77]. Given that we observe a similar moderating influence of sleep duration on brain maturation for both school days and free days, our findings suggest that the total number of hours of sleep is the critical factor. This implies that prioritizing adequate sleep may represent a key behavioral intervention for promoting healthy brain development.

In contrast to two previous cross-sectional studies [35, 36] linking sleep duration with hippocampal volume in youth, we found no moderating effect of sleep duration on volume changes in this structure. However, the two previous studies also contradicted each other – one study [36] linked longer parent-reported sleep, while the other [35] associated shorter self-reported sleep with larger hippocampal volume. These discrepancies may arise from differences in methodology, including poor concordance between self- and parent-reported sleep durations [121, 122], the broad age range and cross-sectional design of previous studies, compared to our within-subject analysis based on objective sleep parameters over a shorter time frame. Our modest sample size and the relatively short six-month interval between MRI scans may also account for the absence of an interaction effect for the hippocampus. Future research should prioritize longitudinal neuroimaging data in large samples to advance our understanding of the links between sleep and brain development in humans [38, 123].

### Sleep efficiency

Sleep efficiency, a key indicator of sleep quality, moderated structural changes in the lateral orbitofrontal cortex, the thalamus and the amygdala. To the best of our knowledge, only a few studies have examined the association between sleep efficiency and grey matter volumes in youth, likely due to the challenges inherent in accurately assessing sleep efficiency using questionnaires. In one study of 56 healthy adolescents aged 12 to 18 years [118], the authors found a negative correlation between the sleep disturbances component of the PSQI and multiple regional grey matter volumes. For example, more disrupted sleep was correlated with smaller volumes in the middle orbitofrontal cortex, which is in line with our results pointing to the relevance of this structure for healthy sleep. However, while we used actigraphy to estimate sleep quality, Sung and colleagues conducted a cross-sectional analysis based on one component of the PSQI, two measures that do not necessarily show large overlap [124].

Another study in young adults in which sleep quality was measured by means of the Pittsburgh Sleep Quality Index (PSQI) found sex-specific relationships between poor sleep quality and brain structure, such that poor female sleepers, but not poor male sleepers, showed altered grey matter volumes in the right parahippocampal gyrus extending to the right hippocampus, in the right inferior parietal lobule and in the right inferior temporal gyrus [125]. The authors suggested that the observed effect for females may be explained by sex-specific hormones that impact sleep promoting circuits and sleep behavior. This study highlights the importance of examining sex-related effects that may add to the understanding of the relationship between sleep and brain maturation.

Shared genetic factors that influence both sleep efficiency and brain maturation may in part account for the links between sleep quality, brain development, and mental health. Support for this conjecture comes from genome wide association studies [126] which point to overlapping genes for sleep difficulties such as insomnia (typically subjectively assessed) and psychiatric conditions (e.g., depression) in which regions such as the orbitofrontal cortex are impacted [127, 128]. Regardless of whether the same genes code for sleep efficiency and brain maturation or other mechanisms are at play, our findings have clear implications – disruptions of sleep may impact the maturation of emotional and reward-related circuits of the brain, potentially creating a vicious loop by which poor sleep and brain functioning reciprocally interact.

### Sleep midpoint

Our findings demonstrate that circadian timing, as indexed by sleep midpoint, moderates changes in cortical volume within the two subcortical regions studied – the thalamus and amygdala. We focused on sleep midpoint measured on free days, as structured school days are more influenced by societal constraints rather than reflecting intrinsic circadian rhythms [129]. We have previously shown that sleep midpoint on free days—a time when teens are largely free to choose their sleep schedule—is predominately determined by genetic factors (over 90%). Disrupted circadian patterns are found across a variety of neurodevelopmental [130] and psychiatric disorders [131]. Furthermore, timing is later in those with subclinical psychiatric traits [132] and later circadian timing (“eveningness”) has been recognized as a risk factor for depressive symptoms and substance abuse (reviewed in [133]). Our findings align with evidence suggesting shared genes between circadian timing and subcortical brain structures and provide new insights into the interplay between circadian biology and brain development [134].

### Sleep regularity

In addition to the above dimensions of sleep (sleep duration, quality and timing) we also opted to examine whether sleep regularity moderates brain maturation. Sleep regularity has emerged as an important metric of sleep health, and irregular sleep wake patterns have been linked to a variety of health and performance related outcomes [93, 135]. Emerging evidence also links sleep regularity to both brain structure and function; for instance, more self-reported irregular sleep habits in adolescence have been shown to be associated with decreased volumes in the amygdala and the right hippocampus [41]. We find that sleep regularity moderates brain maturation across most regions examined, though this effect was at times found for free days and at other times for school days.

Interestingly, the amygdala stands out among our ROIs, displaying a distinct pattern of effects related to levels of the moderator – for sleep duration, sleep efficiency and sleep regularity, values above the mean were associated with *fewer* volumetric changes over time compared to lower values of these metrics. We conclude that for the amygdala, indicators of healthy sleep such as higher sleep efficiency and longer sleep duration come with comparatively little volume change. This finding is in line with our hypothesis that the amygdala follows a distinct developmental trajectory compared to the other regions studied because amygdala volumes increase nonlinearly during this period [59, 75], whereas other structures show a nonlinear decline in volume over this period [59, 75, 136]. Overall, we interpret our findings as suggesting that sleep duration, quality, timing and regularity may contribute to brain maturation during adolescence, potentially by supporting the developmental trajectories of grey matter volume.

### Limitations

While our study offers novel insights into the role of sleep in adolescent brain development, several limitations warrant consideration. First, our sample size was small and consisted of participants with generally healthy sleep patterns and limited variability in sleep measures. A larger, more diverse cohort, including individuals with clinical sleep disturbances, may have revealed stronger or broader effects. Additionally, considering our sample size, we had to limit the number of statistical tests conducted. For this reason, we focused on seven predefined ROIs, however, we may have potentially overlooked other important brain regions. In a similar vein, we acknowledge that we concatenate the data from weekend sleep and sleep on holidays, that is, we treat non-contiguous data from multiple weekends or different periods of vacation as consecutive days and summarize them under the label of free days. This was done because increasing the number of tests conducted was not deemed viable given the small sample size and associated limitations in statistical power. Our small sample size may also have limited our ability to detect sex differences in regional grey matter volumes in structures such as the amygdala and the hippocampus [76, 137]. A larger sample in conjunction with a broader age range may have revealed sex differences in brain volumes. Furthermore, the ICV normalization approach we used (proportion method) may have masked sex differences [7, 110, 137–141]. In addition, we note that this study focused on structural MRI and grey matter volumes only, and we did not include white matter volume, functional connectivity, cortical thickness [35], cortical gyrification [142] which have all been linked to adolescent sleep [143–145].

Notably, in addition to finding no sex differences in brain volumes and sleep behavior in our sample, we also couldn’t detect any systematic variation in sleep behavior across different pubertal stages. We suspect that the absence of puberty-related differences in any of our investigated sleep variables may, again, be attributable to our modest sample size as well as our focus on early adolescence. We hypothesize that differences in sleep behavior may have emerged with a larger sample including participants with more advanced pubertal timing, given that puberty-related differences may emerge only at later stages of puberty [146]. Participants in this study were investigated over six months, however, a longer interval between baseline and follow-up assessment might have been ideal to capture changes in brain volume. The choice of six months was based on the feasibility of asking early adolescents to wear an actigraph daily to obtain an objective measure of sleep/wake duration. Furthermore, while the wearables used in our study showed good sensitivity and accuracy when compared to polysomnography in a sample of healthy adolescents [83], they are prone to an overestimation of TST and SE and an underestimation of WASO and SOL [84, 147]. Therefore, actigraphy data need to be understood as an approximation rather than a perfect reflection of sleep behavior. Under consideration of the six-months interval during which actigraphy data was collected, the recommended concurrent use of sleep diaries [148, 149] to improve the detection of sleep periods was not feasible. However, we improved the estimation of sleep parameters by instructing participants to use event markers at lights out and lights on, which provides information on sleep behavior beyond automatic sleep detection [150–152]. While here, we focused on four individual aspects of sleep health assessed by means of actigraphy, future studies may advance our understanding of how sleep shapes brain development by combining subjective multidimensional sleep health metrics (e.g., RU-SATED; Buysse, 2014 [153]) with objective sleep parameters. Such an approach may help disentangle the impact of subjective and objective sleep parameters and elucidate how these measures uniquely contribute to brain maturation. Also, we note that despite not finding significant changes in brain volume within this period, it is still possible for our sleep variables to modify the associations of volumes over time.

While our study results suggest that sleep may moderate brain development in early adolescence, no causal inferences about the role of sleep or the mechanisms at play with regards to sleep influencing developmental trajectories can be drawn from our data. Moreover, the participants recruited in this study were not fully independent from one another, as the sample consisted of identical and fraternal twins – meaning that the pairs of participants shared at least 50% of their genes. Of note, changes to specific brain volumes have been shown to be heritable [154–156], however, heritability estimates are modest (h^2^ ~ 0.20 - 0.50). Therefore, while our results may have been more pronounced with a sample of fully independent participants, it seems unlikely that the inclusion of identical twins had a large influence on our findings.

## Conclusion: Improving Sleep to Support Brain Development

The American Academy of Pediatrics has labeled insufficient sleep among adolescents as a “public health epidemic” [157] and the adverse consequences of sleep loss for mental well-being are well-documented. Our results add to this important discussion by highlighting sleep’s potential role in brain maturation during adolescence. Our findings point to the possibility that protecting sleep time may be critical for ensuring optimal brain development. While genetic factors influence sleep, numerous environmental factors place adolescents at risk for sleep disruptions. A well-documented example is the misalignment between the biological delay in adolescent sleep timing, resulting in a tendency for late bedtimes, and environmental pressures, such as early school start times, which collectively result in shortened sleep duration [158]. One effective strategy that may promote sufficient sleep in adolescents is parental bedtime setting, which has been associated with increased sleep duration [159–161]. Thus, parents can have a positive impact on brain maturation by ensuring that teens get adequate sleep. For adolescents with sleep difficulties and disrupted circadian rhythms, several effective sleep-based behavioral interventions exist. Prominent among them are cognitive behavioral therapy for insomnia or bright light therapy to advance sleep timing [162–165]. Extending beyond the social structures of families, societal-level interventions—such as delaying school start times—represent a highly impactful strategy. This underscores that promoting healthy sleep in adolescents should not merely be an individual or familial responsibility, but a public health priority.

## Supplementary Material

Supplementary_Materials_Figures_S1-S7_SLEEP_16_01_2026_zsag011

## Data Availability

Anonymized processed data supporting the findings of this study can be shared upon request to the corresponding author and pending ethics approval.

## Notes

Institutions where the work was performed: University Hospital of Child and Adolescent Psychiatry and Psychotherapy, University of Bern, Bern, Switzerland; Translational Research Center, University Hospital of Psychiatry and Psychotherapy, University of Bern, Bern, Switzerland; Center for MR Research, University Children's Hospital Zurich, University of Zurich, Zurich, Switzerland
