# Supplementary material for: Sleep as a moderator of adolescent brain development—evidence from a longitudinal MRI study
Source: Sleep. 2026 Jan 21;49(6):zsag011. doi: 10.1093/sleep/zsag011 (PMC13266549; doi:10.1093/sleep/zsag011)
Supplement: Supplementary_Materials_Figures_S1-S7_SLEEP_16_01_2026_zsag011 [file Supplementary_Materials_Figures_S1-S7_SLEEP_16_01_2026_zsag011.pdf]

Supplementary Materials

Sleep as a moderator of adolescent brain development – evidence from a longitudinal MRI study

Salome Wild [1,2,3], Andrea Inderkum[4], Daniela Rupp[1,5], Andjela Markovic[1,3,6], Chiara E.G. Castiglione[1,3], Christoph Hamann[7], Kristina Adorjan[3], Michael Kaess[1,8], Ruth L. O'Gorman Tuura[9,10,11], and Leila Tarokh [1,3]

[1]University Hospital of Child and Adolescent Psychiatry and Psychotherapy, University of Bern, Bern, Switzerland*

[2]Graduate School for Health Sciences, University of Bern, Switzerland

[3]Translational Research Center, University Hospital of Psychiatry and Psychotherapy, University of Bern, Bern, Switzerland*

[4]Institute of Pharmacology and Toxicology, University of Zurich, Zurich, Switzerland

[5]Bern University of Teacher Education, Bern, Switzerland

[6]Department of Psychology, University of Fribourg, Fribourg, Switzerland

[7]Division of Child and Adolescent Psychiatry and Psychosomatic Medicine, Department of Pediatrics, Inselspital, Bern University Hospital, University of Bern, Bern, Switzerland

[8]Department of Child and Adolescent Psychiatry, Centre for Psychosocial Medicine, University Hospital Heidelberg, Heidelberg, Germany

[9]Center for MR Research, University Children's Hospital Zurich, University of Zurich, Zurich, Switzerland*

[10]University of Zurich, Zurich, Switzerland

[11]Children's Research Centre, University Children's Hospital Zurich, Zurich, Switzerland

* Institutions where the work was performed.

Corresponding author:

Leila Tarokh, PhD

Translational Research Center, University Hospital of Psychiatry and Psychotherapy, University of Bern

Bolligenstrasse 111

3000 Bern, Switzerland

Email: leila.tarokh2@unibe.ch

This file includes: Supplementary figures S1 - S7

Figure S1. Conditional effects for significant interactions**:** Total Sleep Time on school days as a moderator of brain volume change

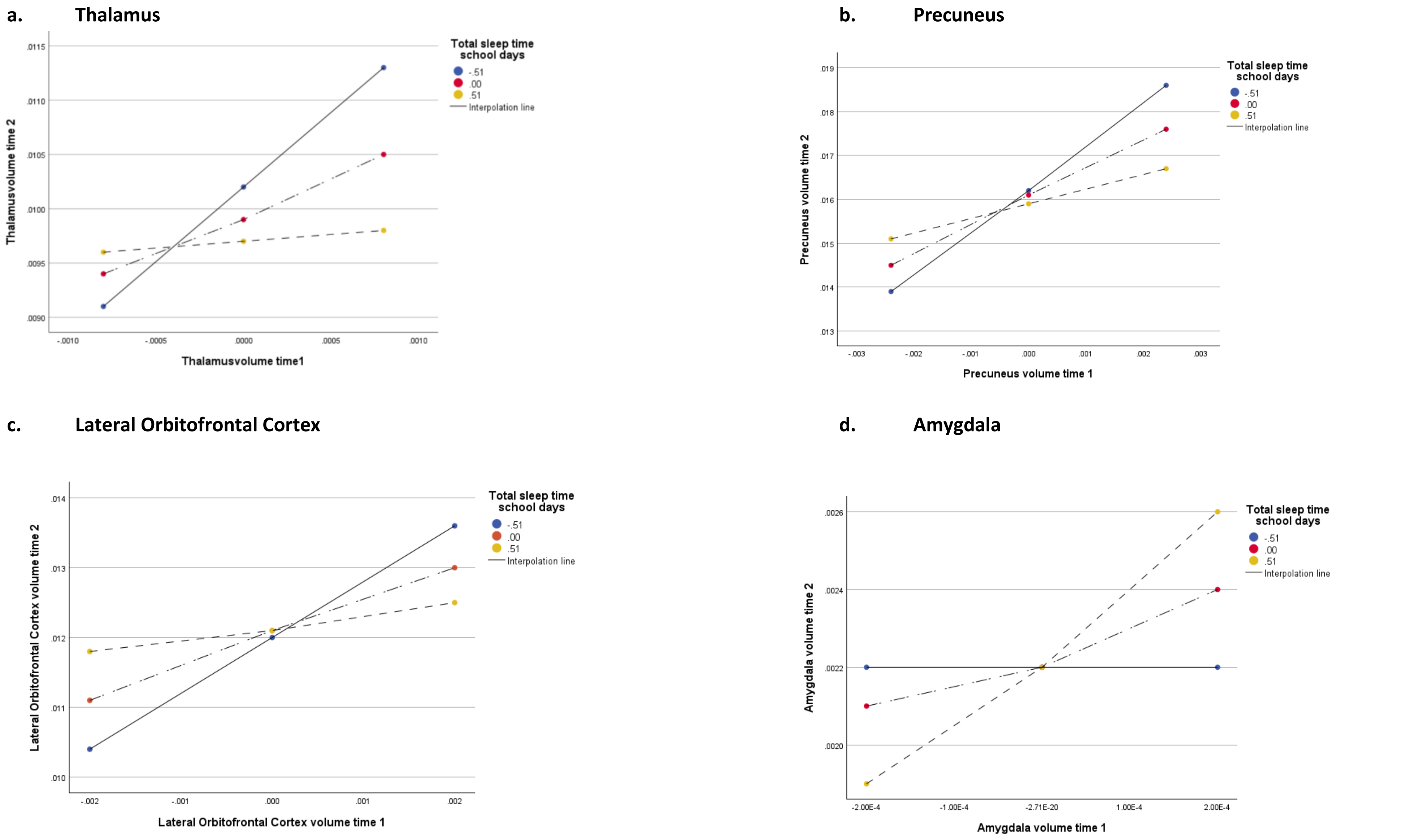

Simple slopes for grey matter volumes at mean values of Total Sleep Time (TST) on school days and at ± 1 SD from the mean, illustrating the examination of conditional effects. The associations between time 1 and time 2 volumes are shown for mean TST (i.e., red dots), corresponding to 8.18 hours of sleep. The associations between time 1 and time 2 volume for above and below mean TST are depicted in the simple slopes connecting the yellow and blue dots respectively, with above mean TST corresponding to 8.69 h and below mean TST to 7.67 hours of sleep.

Figure S2. Conditional effects for significant interactions: Total Sleep Time on free days as a moderator of brain volume change

a. Thalamus

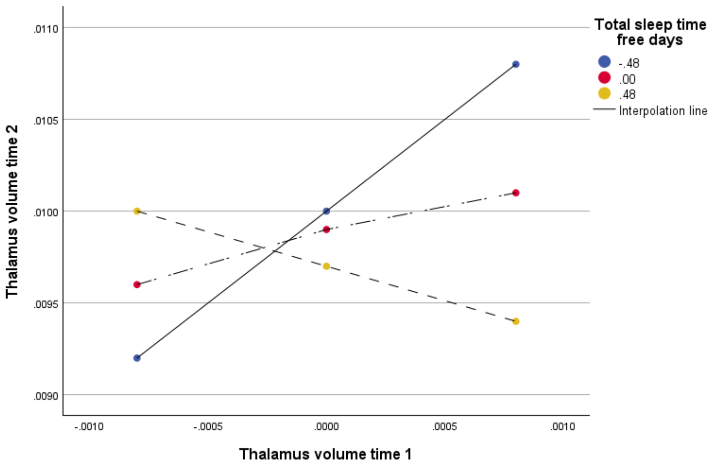

b. Lateral Orbitofrontal Cortex

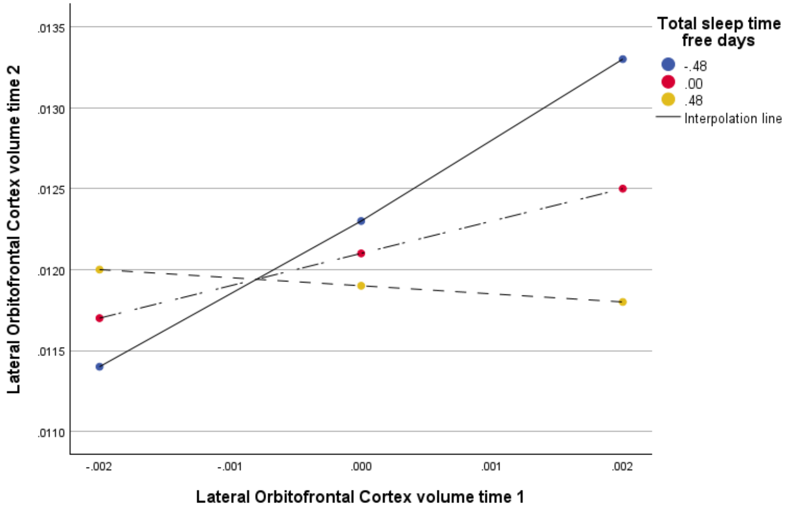

c. Medial Orbitofrontal Cortex

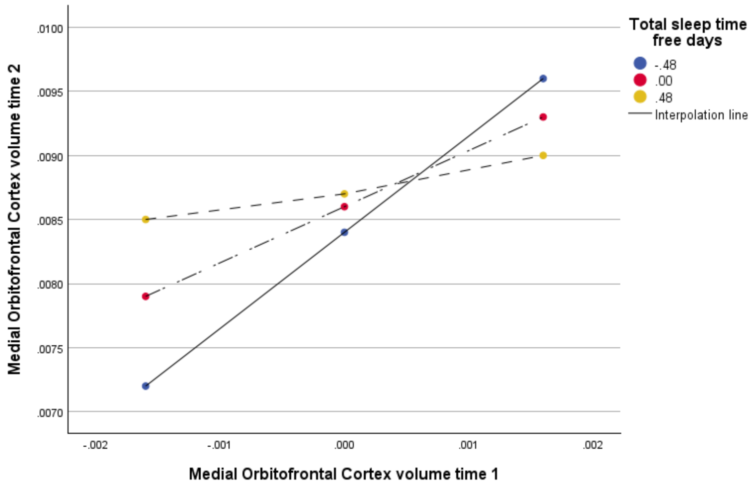

d. Precuneus

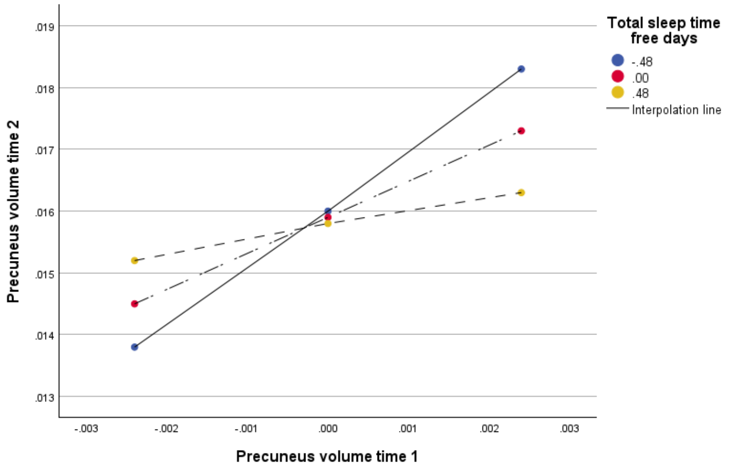

Simple slopes for grey matter volumes at mean values of Total Sleep Time (TST) on free days and at ± 1 SD from the mean, illustrating the examination of conditional effects. The associations between time 1 and time 2 volumes are shown for mean TST (i.e., red dots), corresponding to 8.56 hours of sleep. The associations between time 1 and time 2 volume for above and below mean TST are depicted in the simple slopes connecting the yellow and blue dots respectively, with above mean TST corresponding to 9.05 h and below mean TST to 8.09 hours of sleep.

Figure S3. Conditional effects for significant interactions: Sleep efficiency on school days as a moderator of brain volume change

a. Thalamus

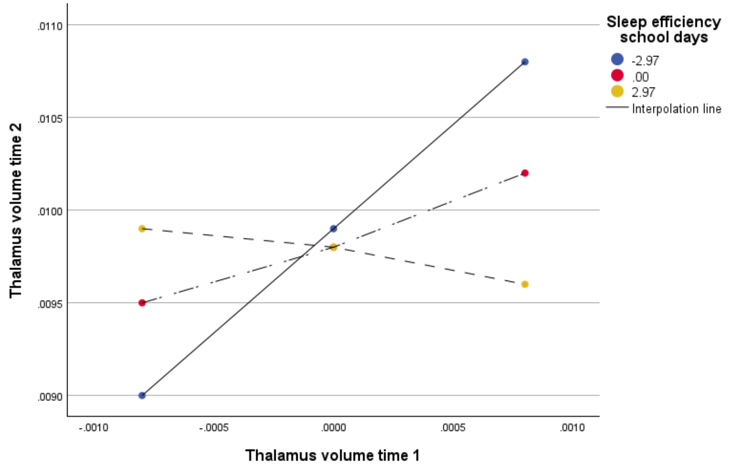

b. Lateral Orbitofrontal Cortex

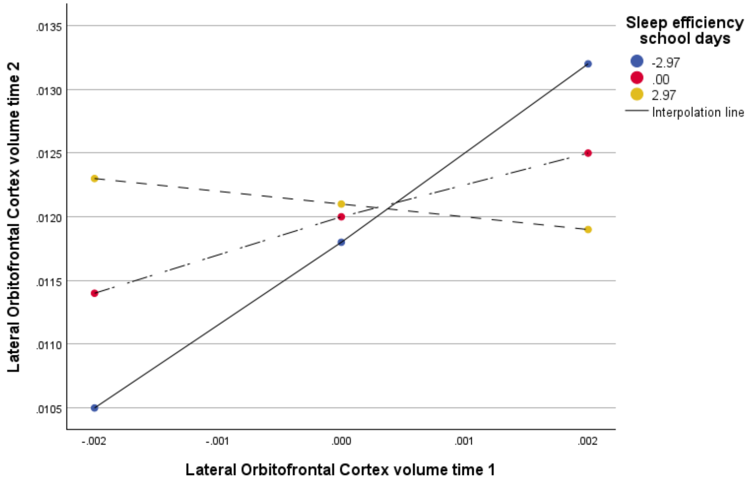

c. Amygdala

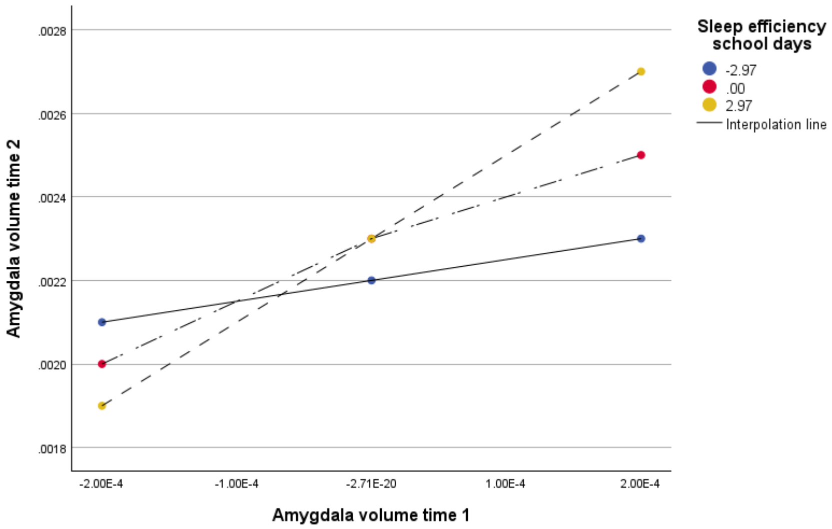

Simple slopes for grey matter volumes at mean values of sleep efficiency on school days and at ± 1 SD from the mean, illustrating the examination of conditional effects. The associations between time 1 and time 2 volumes are shown for mean sleep efficiency (i.e., red dots), corresponding to 90.87%. The associations between time 1 and time 2 volume for above and below mean sleep efficiency are depicted in the simple slopes connecting the yellow and blue dots respectively, with above mean sleep efficiency corresponding to 93.83%, and below mean sleep efficiency to 87.90%.

Figure S4. Conditional effects for significant interactions: Sleep efficiency on free days as a moderator of brain volume change

a. Thalamus

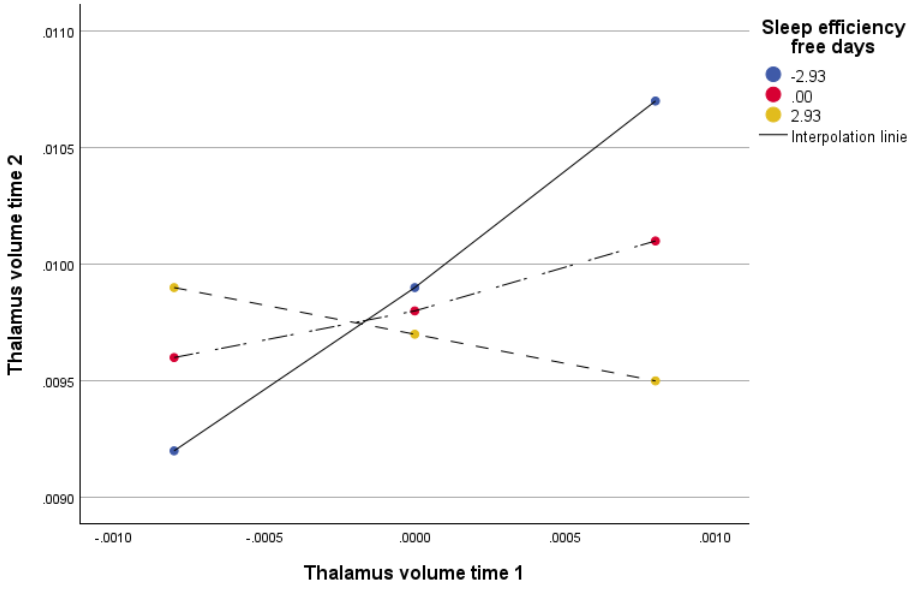

b. Lateral Orbitofrontal Cortex

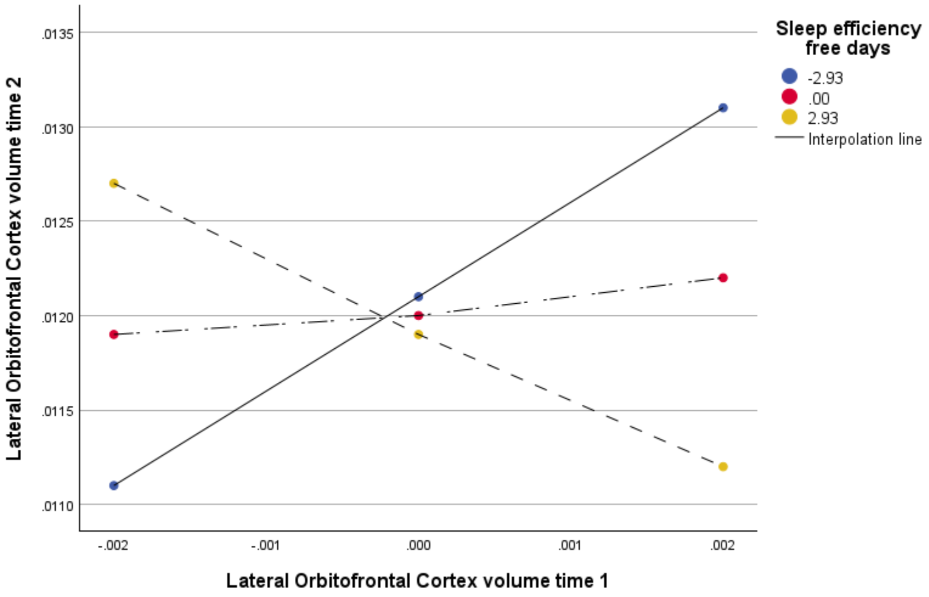

Simple slopes for grey matter volumes at mean values of sleep efficiency on free days and at ± 1 SD from the mean, illustrating the examination of conditional effects. The associations between time 1 and time 2 volumes are shown for mean sleep efficiency (i.e., red dots), corresponding to 91.26%. The associations between time 1 and time 2 volume for above and below mean sleep efficiency are depicted in the simple slopes connecting the yellow and blue dots respectively, with above mean sleep efficiency corresponding to 94.19%, and below mean sleep efficiency to 88.33%.

Figure S5. Conditional effects for significant interactions: Sleep midpoint on free days as a moderator of brain volume change

a. Thalamus

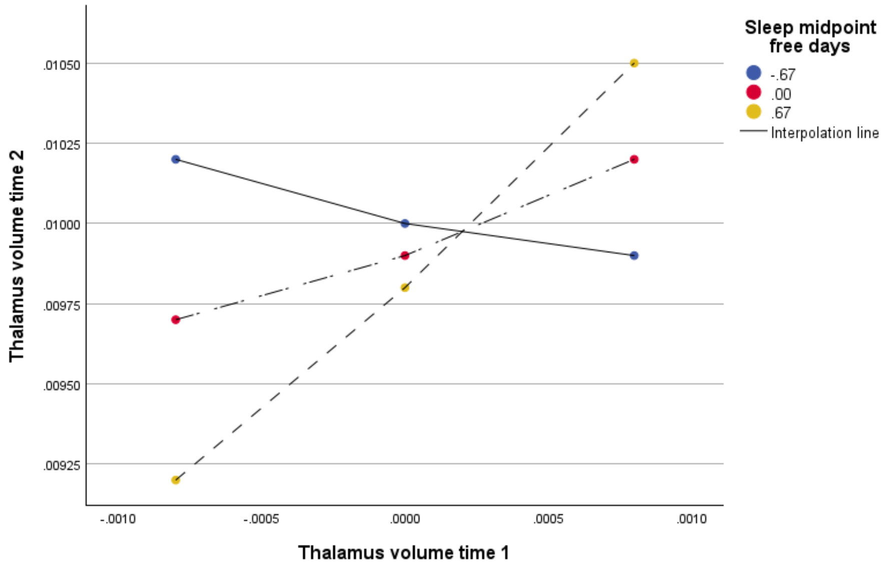

b. Amygdala

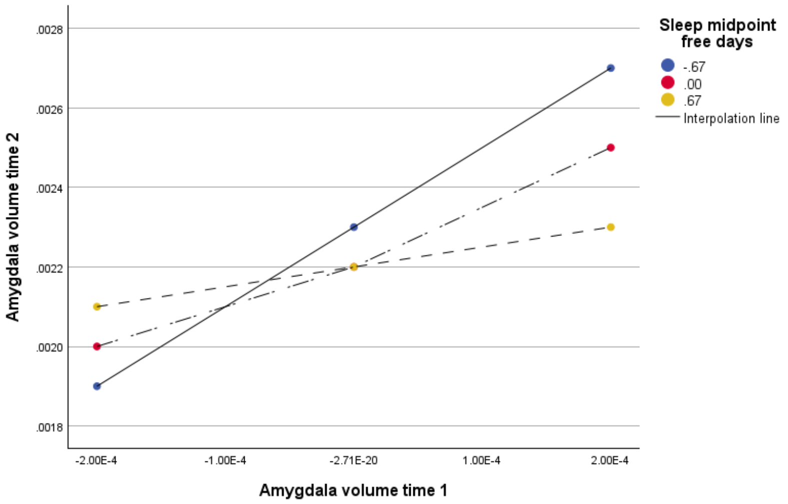

Simple slopes for grey matter volumes at mean values of sleep midpoint on free days and at ± 1 SD from the mean, illustrating the examination of conditional effects. The associations between time 1 and time 2 volumes are shown for mean sleep midpoint (i.e., red dots), corresponding to 03:48 am. The associations between time 1 and time 2 volume for above and below mean sleep midpoint are depicted in the simple slopes connecting the yellow and blue dots respectively, with above mean sleep midpoint corresponding to 4:29 am, and below mean sleep midpoint to 3:08 am.

Figure S6. Conditional effects for significant interactions: Sleep regularity on weekdays as a moderator of brain volume change

a. Thalamus

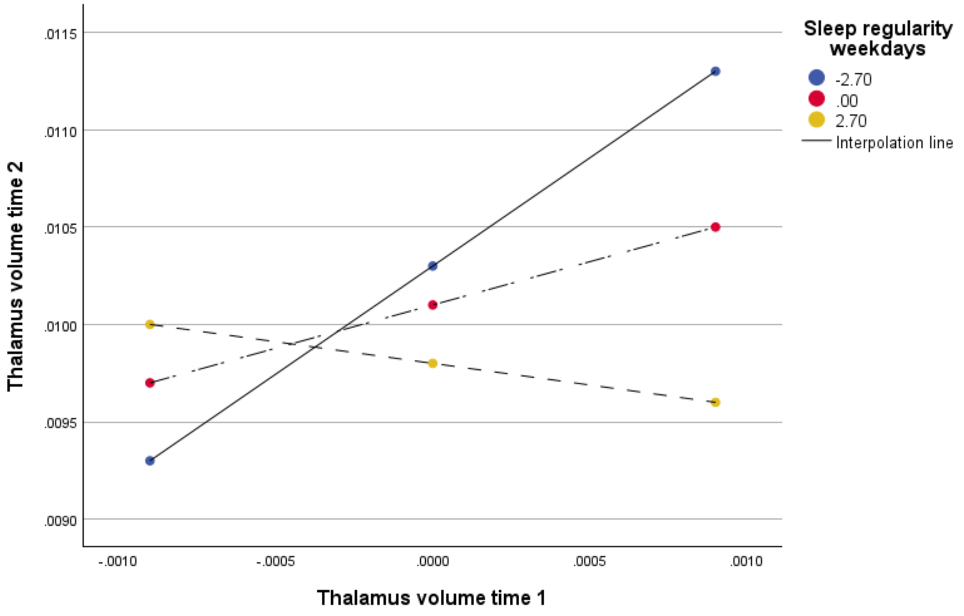

b. Amygdala

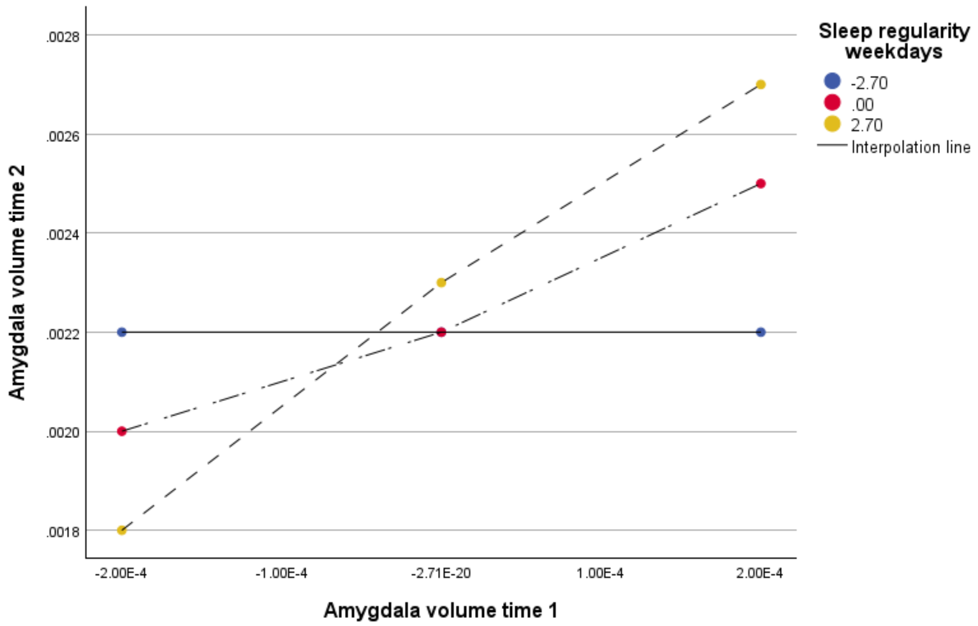

Simple slopes for grey matter volumes at mean values of sleep regularity on weekdays (as measured by SRI) and at ± 1 SD from the mean, illustrating the examination of conditional effects. The associations between time 1 and time 2 volumes are shown for mean sleep regularity (i.e., red dots), corresponding to sleep regularity of 91.18. The associations between time 1 and time 2 volume for above and below mean sleep regularity are depicted in the simple slopes connecting the yellow and blue dots respectively, with above mean sleep regularity corresponding to 93.88, and below mean sleep regularity to 88.48.

Figure S7. Conditional effects for significant interactions: Sleep regularity on free days as a moderator of brain volume change

a. Medial Orbitofrontal Cortex **b. Precuneus**

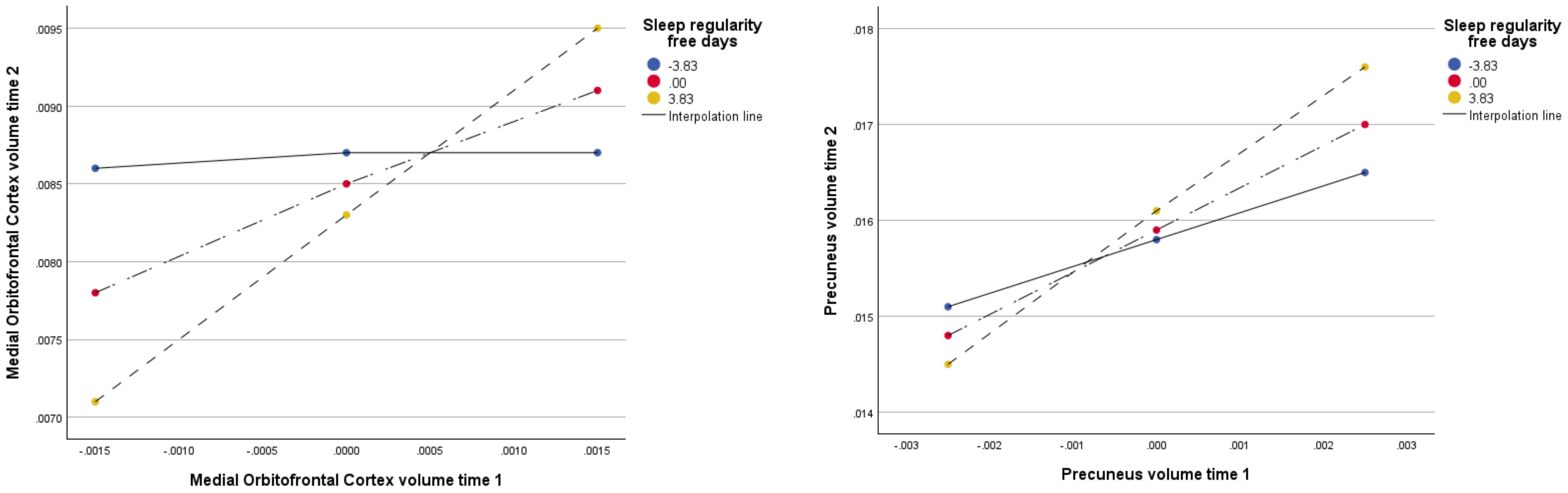

Simple slopes for grey matter volumes at mean values of sleep regularity on free days (as measured by SRI) and at ± 1 SD from the mean, illustrating the examination of conditional effects. The associations between time 1 and time 2 volumes are shown for mean sleep regularity (i.e., red dots), corresponding to sleep regularity of 84.25. The associations between time 1 and time 2 volume for above and below mean sleep regularity are depicted in the simple slopes connecting the yellow and blue dots respectively, with above mean sleep regularity corresponding to 88.07, and below mean sleep regularity to 80.40.
